# A Deep Learning Model to Predict Breast Implant Texture Types Using Ultrasonography Images: Feasibility Development Study

**DOI:** 10.2196/58776

**Published:** 2024-11-05

**Authors:** Ho Heon Kim, Won Chan Jeong, Kyungran Pi, Angela Soeun Lee, Min Soo Kim, Hye Jin Kim, Jae Hong Kim

**Affiliations:** 1 Department of Biomedical Informatics Medical School of Yonsei University Seoul Republic of Korea; 2 3Billion, Inc Seoul Republic of Korea; 3 Quantic EMBA Washington, DC United States; 4 Korean Society of Breast Implant Research Seoul Republic of Korea; 5 The W Clinic Seoul Republic of Korea

**Keywords:** breast implants, mammoplasty, ultrasonography: AI-assisted diagnosis, cshell surface topography, artificial intelligence, deep learning, machine learning

## Abstract

**Background:**

Breast implants, including textured variants, have been widely used in aesthetic and reconstructive mammoplasty. However, the textured type, which is one of the shell texture types of breast implants, has been identified as a possible etiologic factor for lymphoma, specifically breast implant–associated anaplastic large cell lymphoma (BIA-ALCL). Identifying the shell texture type of the implant is critical to diagnosing BIA-ALCL. However, distinguishing the shell texture type can be difficult due to the loss of human memory and medical history. An alternative approach is to use ultrasonography, but this method also has limitations in quantitative assessment.

**Objective:**

This study aims to determine the feasibility of using a deep learning model to classify the shell texture type of breast implants and make robust predictions from ultrasonography images from heterogeneous sources.

**Methods:**

A total of 19,502 breast implant images were retrospectively collected from heterogeneous sources, including images captured from both Canon and GE devices, images of ruptured implants, and images without implants, as well as publicly available images. The Canon images were trained using ResNet-50. The model’s performance on the Canon dataset was evaluated using stratified 5-fold cross-validation. Additionally, external validation was conducted using the GE and publicly available datasets. The area under the receiver operating characteristic curve (AUROC) and the area under the precision-recall curve (PRAUC) were calculated based on the contribution of the pixels with Gradient-weighted Class Activation Mapping (Grad-CAM). To identify the significant pixels for classification, we masked the pixels that contributed less than 10%, up to a maximum of 100%. To assess the model’s robustness to uncertainty, Shannon entropy was calculated for 4 image groups: Canon, GE, ruptured implants, and without implants.

**Results:**

The deep learning model achieved an average AUROC of 0.98 and a PRAUC of 0.88 in the Canon dataset. The model achieved an AUROC of 0.985 and a PRAUC of 0.748 for images captured with GE devices. Additionally, the model predicted an AUROC of 0.909 and a PRAUC of 0.958 for the publicly available dataset. This model maintained the PRAUC values for quantitative validation when masking up to 90% of the least-contributing pixels and the remnant pixels in breast shell layers. Furthermore, the prediction uncertainty increased in the following order: Canon (0.066), GE (0072), ruptured implants (0.371), and no implants (0.777).

**Conclusions:**

We have demonstrated the feasibility of using deep learning to predict the shell texture type of breast implants. This approach quantifies the shell texture types of breast implants, supporting the first step in the diagnosis of BIA-ALCL.

## Introduction

Breast implants have been developed for aesthetic and reconstructive mammaplasty since 1962. The first textured breast implant was developed in 1968 to prevent capsular contracture after aesthetic or reconstructive implant-based mammaplasty [1,2]. Engraving and embossing types of textured implants have also been used in anatomical breast implants for natural shape. Since the diagnosis of the first case of breast implant–associated anaplastic large cell lymphoma (BIA-ALCL) in 1997 by Dr. Keech, a total of 1264 cases of BIA-ALCL, including 59 deaths, have been reported by the US Food and Drug Administration (FDA), according to a recent update as of June 30, 2023 [3,4]. Since the first case of BIA-ALCL, numerous investigations have been conducted to examine its etiology, prevalence rates, and clinical characteristics.

Several studies have demonstrated that the topography of a textured breast implant shell surface is associated with BIA-ALCL [5-11]. Classified as a rare T-cell lymphoma, BIA-ALCL is nevertheless a significant concern in the context of breast augmentation and reconstruction surgeries, with documented cases of mortality. BIA-ALCL is often treatable when detected early, underscoring the critical importance of timely and accurate diagnosis [6,9-11]. A recent study found that prophylactic replacement can be indicated in asymptomatic, risk-stratified patients to reduce the risk of BIA-ALCL [12]. Identifying the inserted breast implant shell surface topography is the first step for diagnosing BIA-ALCL in follow-up breast examination and replacement cases. If a patient has a history of primary aesthetic or reconstructive mammaplasty utilizing a smooth-type breast implant, there is generally no cause for concern regarding BIA-ALCL.

Nevertheless, a substantial number of patients may not be aware of the specific type of breast implant shell inserted during surgery after a long period of time, and medical records, especially within private clinics, may not be well preserved. Additionally, there are flaws in government policy regulating medical devices in South Korea [13]. As the medical community deepens its understanding of the complexities of this condition, the significance of diagnosing the surface topography of breast implant shells becomes increasingly apparent. Traditional diagnostic methods for assessing breast implants have relied heavily on subjective human evaluations. Mammograms and magnetic resonance imaging (MRI) exams, which are used to monitor breast implant–related complications, cannot identify the implant shell surface topography. Only ultrasonography can identify the inserted implant shell surface topography [14,15].

However, ultrasonography also has many limitations. The generalizability of the results may be limited due to the real-time and operator-dependent nature of ultrasonography, which may result in inter- and intraobserver variability. Although artificial intelligence (AI) is useful in overcoming these limitations of ultrasonography, we conducted this study to determine whether it is useful in distinguishing breast implant shell surface topography using our algorithm. Additionally, the development of AI programs to accurately diagnose breast implant shell surface topography holds promise in revolutionizing early detection and management strategies for BIA-ALCL. The development of AI algorithms aims to eliminate subjectivity and provide a more accurate and standardized approach to identifying the breast implant shell surface topography as textured and smooth types.

## Methods

### Study Design

In this study, we retrospectively collected anonymous and deidentified medical records containing information on implant shell types, ultrasonographic images, and demographic characteristics. We built multiple datasets as follows: Canon dataset (D1), GE dataset (D2), ruptured implant dataset (D3), no implant image dataset (D4), and publicly available dataset (D5). We used the Canon dataset (D1) for training, interval validation, and testing, and we used the GE and publicly available datasets (D2 and D5) for external validation. The ruptured implant and no implant image datasets (D3 and D4) were also used as out-of-distribution (OOD) datasets to identify model interpretation.

First, the Canon and GE datasets (D1 and D2) included the ultrasonography images with medical data generated from patients who underwent aesthetic or reconstructive implant-based mammaplasty without implant rupture at a single institution in South Korea. All patients underwent both breast cancer examination and ultrasonography-assisted examination at the institution. Ultrasonography-assisted examinations were conducted with an Aplio i600 (Canon Medical System) system with a 7-18 MHz linear transducer (General Electric LOGIQ E10). These retrospective data were confirmed by a surgeon between August 31, 2017, and November 31, 2022. We obtained the ultrasonography images with medical data from 1043 patients (Multimedia Appendix 1). Multiple ultrasonography images from each patient were captured to assess the implant shell types and saved in a Picture Archiving and Communication System (PACS)–rendered JPEG format. We retained unique images for model development by checking 128-bit MD5 hash algorithms to rule out data leakage between the training and testing datasets. A breast surgeon with 14 years of breast implant ultrasonography experience labeled all the ultrasonographic images of shell surface topography.

Our problem divided shell surface topography into textured and smooth types. The smooth type included the microtextured type as a conventional clinical classification [15]. Microtextured types show almost the same shell surface topography as smooth types in high-resolution ultrasonography and light microscopy [15]. As some retrospective data were not stored in a Digital Imaging and Communications in Medicine (DICOM) format, we only used the centered PACS-rendered image (Table 1), discarding the top 12%, bottom 10%, and left and right 7% of pixels.

**Table 1:** Eligible ultrasonography datasets.

Dataset name	Device	Objective	Shell integrity	Shell surface topography (N=19,502), n	
				Textured	Smooth
Canon (D1)	Canon	Training, validation, and testing	Intact	2420	14,976
GE (D2)	GE	External validation	Intact	113	1844
Ruptured implant (D3)	Canon	OOD^a^	Ruptured	101	30
Without implant (D4)	Canon	OOD	N/A^b^	N/A^c^	N/A^c^
Publicly available (D5)	Heterogenous	External validation	Intact	11	7

^a^OOD: out-of-distribution.

^b^N/A: not applicable.

^c^n=338.

Second, 131 ultrasonography images of the ruptured implant dataset (D3) were collected using the Canon Aplio i600. Because of the damaged shell integration, the implant shell type would be less easily identified from ultrasonography images. We used these images as an OOD dataset to identify the model’s ability to estimate its uncertainty for the 2 types of shells. Third, 338 ultrasonography images without implants were also captured and used as an OOD dataset to determine the transparency of our model by estimating uncertainty (D4). Finally, we constructed a publicly available dataset for external validation by searching for ultrasonography images using the following keywords: “breast implant ultrasound” and “breast implant ultrasonography” (D5).

### Model Development

Convolutional neural networks (CNNs) were used to scale the parameter sizes to achieve high performance. This feasibility study used an off-the-shell CNN architecture originally designed for natural images, ResNet-50, composed of 50 layers as the backbone [16]. To speed up the proof of concept, we chose a lightweight model instead of models with a large number of parameters, such as Vision transformer or SwinTransformer, which require expensive computational costs with a large amount of data due to the lack of inductive bias [17,18].

We trained our model by conducting transfer learning on the pretrained ResNet-50, which learned ImageNet classification. Then, we replaced the classifier layer of ResNet-50, which has a 1000D vector for multiclass, with a binary classifier layer to return a 2D vector for shell surface topology as smooth or textured type. Weighted binary cross-entropy was used as the objective function for parameter optimization, which effectively trains the model by penalizing incorrect predictions of the minor class due to class imbalance between shell types (the textured type being a minor class). The weight in the weighted binary cross-entropy for the minor class was calculated with the inversed ratio of the minor class in the training dataset.

Cropped, PACS-rendered images were preprocessed, being resized to 224×224 pixels using bilinear interpolation. The images in the training dataset were fed into the model without any augmentation. The deep learning model was trained with an Adam optimizer, with a learning rate 0.0001 and a batch size 32. The total number of training epochs was set to 20; however, the actual number of epochs was reduced due to early stopping, which was triggered when the validation loss did not improve for 7 consecutive epochs (patience=7).

### Performance Evaluation and Model Interpretation.

**Figure 1:**
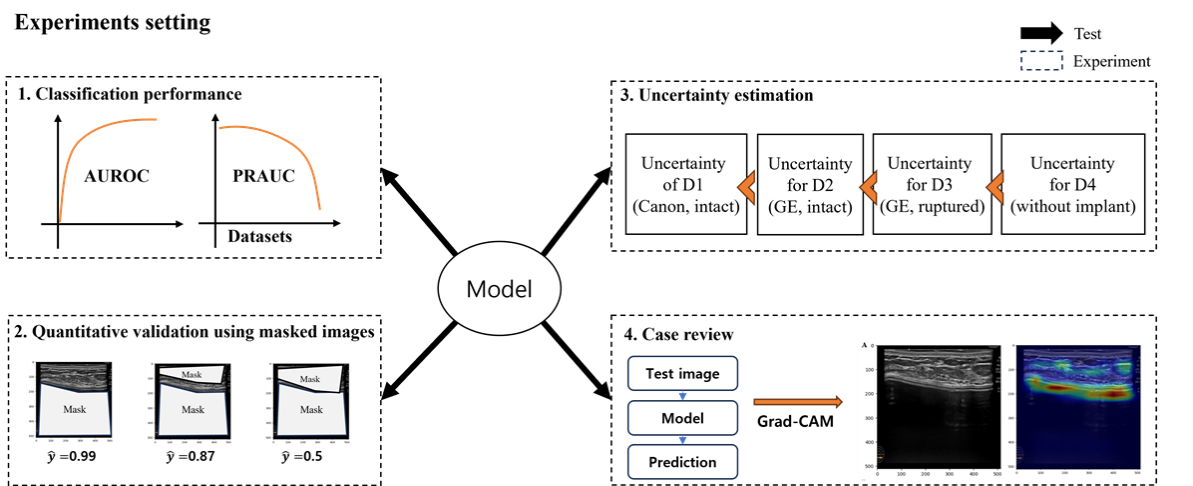
Illustration of the 4 experimental settings. AUROC: area under the receiver operating characteristic curve; Grad-CAM: Gradient-weighted Class Activation Mapping; PRAUC: area under the precision-recall curve.

We evaluated our model with four experiments: (1) classification performance using multiple datasets, (2) quantitative validation using masked images, (3) uncertainty estimation for multiple datasets, and (4) post hoc explainable interpretation (Figure 1).

First, to report our model transparently, we performed two types of validation: (1) stratified cross-validation and (2) external validation with both the GE dataset and publicly available ultrasonography images (D2 and D3). A stratified 5-fold cross-validation was conducted to identify generalized performance in the Canon dataset due to class imbalance between smooth and textured shells. Data split was performed by stratified random splitting of ultrasonography images into training (60%), validation (20%), and test (20%) datasets with shell surface topology labels. We evaluated the area under the curve (AUC) with different cutoffs for the receiver operating characteristic (ROC) curve and precision-recall (PR) curve. Also, we conducted an external validation set to reduce latent bias with publicly available ultrasonographic images. We also identified the AUC of both the ROC and PR curves with these data.

Second, we used an explainable AI (XAI) approach to determine whether our model accurately classified the implant shell types from the features of echogenicity or layers from ultrasonography images and no other unexpected factors by assessing the classification performance according to the masking part of the image. For the quantitative validation, we hypothesized that the important pixels of the image that distinguished the type of implant were on the layers of the implant. In addition, we also hypothesized that there would be no performance degradation if some nonlayered pixels were erased. Therefore, we calculated the pixel importance using Gradient-weighted Class Activation Mapping (Grad-CAM), a method to quantify a pixel’s contribution to the classification [19]. Both AUROC and PRAUC were calculated by removing 10% of the least-contributing pixels from the total number of pixels in the image and replacing them with zeros.

Third, we calculated the Shannon entropy for the uncertainty estimation to estimate the predictive uncertainty for each image in the OOD dataset [20,21]. Entropy ranges from 0 to 1, with a larger value indicating greater predictive uncertainty. We also hypothesized that entropies in the ruptured implant dataset (D3) would be larger than those in the test set because our model was only trained on ultrasonography images from patients without damaged shell integrity. Furthermore, we hypothesized that the entropies in images without breast implants (D4) would be larger than those in the ruptured implant dataset (D3). Our model was only trained on ultrasonographic features from the implant image dataset to classify the shell types.

Finally, we used the Grad-CAM technique to gain further insight into the model’s decision-making processes. This interpretative method was used to visually trace and affirm the alignment between the model’s predictive patterns and established medical expertise concerning diagnosing different breast implant shell types. Through Grad-CAM, heatmaps were generated, highlighting the critical regions in the imaging data that influenced the model’s diagnostic predictions, thus ensuring that these insights were consistent with conventional medical knowledge.

### Ethical Considerations

This retrospective study was approved by the Internal Institutional Review Board of the Korea National Institute of Bioethics Policy (P01-202401-01-006), which waived the requirement for informed consent of medical records, including patients’ images and characteristics. All procedures described herein were performed under the 1964 Declaration of Helsinki and its later amendments or comparable ethical standards**.** None of the authors have a financial interest in any products, devices, or drugs mentioned in this paper.

## Results

### Classification Performance for Shell Surface Type

Our model achieved an AUROC of 0.998 and a PRAUC of 0.994 in the Canon dataset (D1; Table 2). From the stratified 5-fold cross-validation, our model showed an average AUROC of 0.98 and a PRAUC of 0.88 in the Canon dataset captured with the Canon ultrasonography device (D1; Multimedia Appendix 2). Although the images were captured with a GE ultrasonography device (D2), our model showed an AUROC of 0.985 and a PRAUC of 0.748. For the publicly available dataset (D5), the model showed an AUROC of 0.909 and a PRAUC of 0.958.

**Table 2:** Performance metrics of classification for each dataset

Metrics	Datasets			
	Canon (D1)	GE (D2)	Ruptured implants (D3)	Publicly available (D5)
AUROC^a^	0.998	0.985	0.995	0.909
PRAUC^b^	0.994	0.748	0.998	0.958

^a^AUROC: area under the receiver operating characteristic curve.

^b^PRAUC: area under the precision-recall curve.

### Quantitative Validation

**Figure 2:**
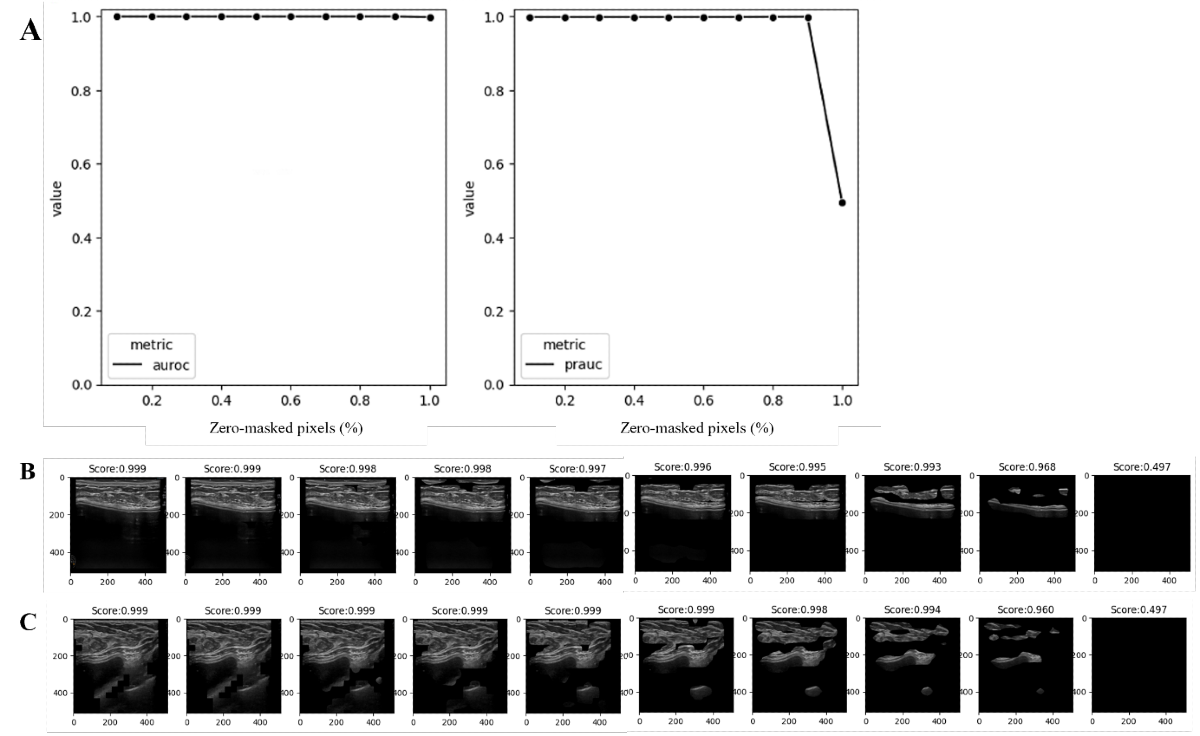
Performance deterioration depends on masking noncontributing pixels to prediction. (A) ROC curve (left) and PR curve (right) for the test dataset in the Canon dataset; (B) textured shell implant prediction in the Canon dataset (D1) by increasing the number of lower-contributing pixels by 10%; (C) textured shell implant prediction in the ruptured implant dataset (D3) by increasing the number of lower-contributing pixels by 10%. AUROC: area under the receiver operating characteristic curve; PR: precision-recall; PRAUC: area under the precision-recall curve; ROC: receiver operating characteristic.

In the quantitative analysis to determine whether our model classifies ultrasonography images by medical knowledge, the model maintained an AUROC of 0.999 when masking up to 90% of the least-contributing pixels for prediction and showed an AUROC of 0.997 when masking 100% of the pixels. However, the PRAUC remained at 0.999 even after masking 90% of the pixels. After that, it decreased to 0.493 when all pixels were masked (Figure 2A). For each individual case, the confidence for the textured shell type remained at 0.993, even when 80% or fewer contributing pixels were masked. When masking 90% of the pixels, model confidence dropped to 0.968 and reached 0.497 when all pixels were masked. Similarly, the model confidence for another case with a textured shell type was maintained at 0.994 until masking 80% of the pixels, decreased to 0.960 when masking 90% of the pixels, and dropped to 0.947 when masking 100% of the pixels (Figure 2B and C).

### Uncertainty Estimation

**Figure 3:**
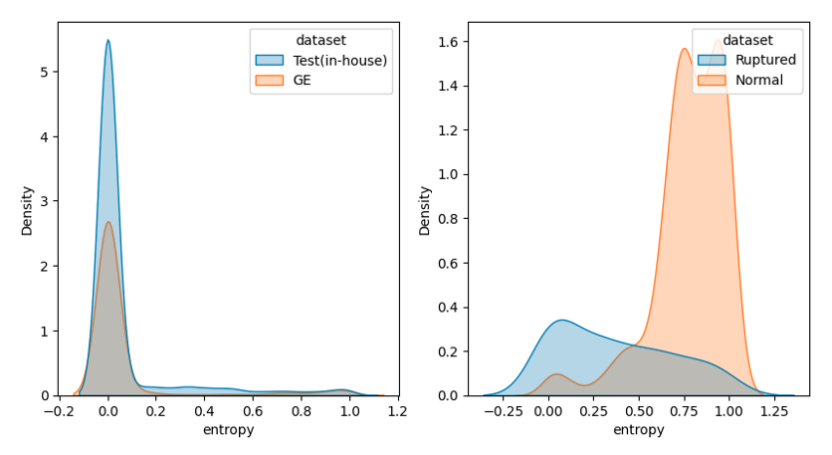
Entropy distribution of prediction from tests in all datasets.

The model did not produce significantly lower entropies for the test dataset in the Canon dataset (D1) than for the external validation set from the GE ultrasonography device (mean 0.072, SD 0.201 vs mean 0.066, SD 0.21; *P*=.35). However, the average entropy for ruptured implant images was significantly higher than for the test dataset in the Canon dataset (mean 0.371, SD 0.318 vs mean 0.072, SD 0.201; *P*<.001). Moreover, the model also predicted a statistically significantly higher entropy for images with breast implants than for ruptured implant images (mean 0.777, SD 0.199 vs mean 0.371, SD 0.318; *P*<.001; Figure 3).

### Individual Case Review

**Figure 4:**
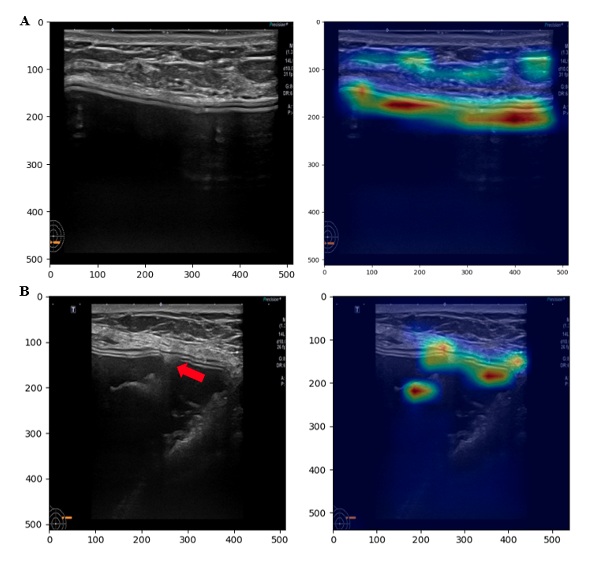
Preprocessed ultrasonography image with Grad-CAM for textured shell type prediction. Heatmap with Grad-CAM was bilinearly interpolated to resize the original image. (A) Intact textured implant image captured with Canon ultrasonography device, and (B) a damaged textured implant image captured with Canon ultrasonography device (the red arrow annotates a shell tear). Grad-CAM: Gradient-weighted Class Activation Mapping.

For a qualitative case review, we sampled 2 ultrasonography images, 1 from the test dataset (Canon, D1) and another from the ruptured implant datasets (D3), captured by the same device. The model provided a model confidence of 0.998 for the textured shell type. In a heatmap with the Grad-CAM score, high values were shown for the textured type at the shell (white horizontal line in Figure 4A). Also, for the image of the ruptured, textured shell implant, the model provided model confidence of 0.664 for it being the textured type. Although this score is higher than the classification threshold (0.5), it is lower than that of the intact, textured shell implant. However, the Grad-CAM score was high in the intact layer adjacent to the ruptured shell area in the heatmap despite the shell being ruptured due to a shell tear (Figure 4B).

## Discussions

### Principal Findings

Identifying breast implant shell types requires ultrasonographic examination, which can have inter- and intraobserver variability. Therefore, the generalizability of the results may be variable, leading to potentially missed diagnoses. However, no quantitative measurement or classification method distinguishes the 2 shell types. This feasibility study demonstrates that deep learning can quantitatively classify breast implant shell types. Also, this study supports the use of echogenicity from the shell layer of breast implants as an important region in classifying shell types. Furthermore, despite using different ultrasonography devices to capture images, our findings provide evidence that the deep learning model can classify the shell types. Moreover, the model exhibited higher uncertainty for ruptured breast implant ultrasonography images and ultrasonography images without an implant than images from the intact shell type classification dataset, suggesting that the model could robustly quantify predictive uncertainty.

### Clinical Application

There are several classifications for breast implant shell surface topography; ISO 14607:2018 is a widely accepted classification [22]. Although there is a lack of standardized breast implant surface classification, high-resolution ultrasonography of shell surface topography can divide the breast implant texture types into textured and smooth [15]. The textured type shows roughness compared with the smooth type in high-resolution ultrasonography (Multimedia Appendix 3) [15]. Identifying the texture type of an inserted implant using ultrasonography without surgery is clinically important because the physician must consider the BIA-ALCL risk induced by the textured-type breast implant in patients, even in patients with no memory of the implant. As an extension of previous research on the feasibility of high-resolution ultrasonography for identifying the breast implant shell surface topography, this study was conducted to develop a deep learning model to predict the breast implant texture types with ultrasonographic images [15]. In ultrasonography, smooth types include microtextured types because microtextured types show almost the same shell surface compared with smooth types.

This method offers a promising way to classify breast implants with respect to the risk of BIA-ALCL, a condition that remains underexplored in current research. Given its rarity and the association of certain texture types with BIA-ALCL, accurate identification of texture type emerges as a critical determinant in risk assessment. Using ultrasonography to identify texture types allows for straightforward identification on ultrasound images, often eliminating the need for additional testing. In addition, the use of deep learning models has the potential to assist patients undergoing breast augmentation or reconstruction, particularly in cases of implant rupture. Given the limited familiarity of radiologists and breast physicians with the diverse landscape of breast implants, including both manufacturers and shell types, the integration of AI in clinical contexts is proving invaluable.

### Reliable AI for Clinical Decision Support

Reliable AI is essential for clinical decision support in the biomedical domain to avoid adverse patient outcomes [23,24]. This study includes multiple experiments on different datasets, such as devices and OOD datasets, to explore the model’s transparency. In AI research for radiology, it was found that deep learning models often showed deteriorated performance in external validation [25]. The study reveals that deep learning models may be vulnerable to medical images from heterogeneous sources due to unseen distribution. To eliminate biased evidence from these findings, we evaluated the model using ultrasonography images from the heterogeneous devices (Figure 1). In addition, this study showed uncertainty in the model’s predictions, with the mean distribution being larger for images taken with the same device, images taken with a different device, images with ruptured implants, and images without implants (Figure 2). This can support the idea that the deep learning model classifies the shell type by learning the ultrasonographic features of breast implant shells. Further, ruptured implant images are consistent with those in the medical field, where determining the shell type of a ruptured implant is difficult due to the damaged surface of the implant (Figure 3). The entropies for ruptured implant images (D4) were higher than those for intact implant images (D1 and D2). This approach provides model confidence that can help clinicians make decisions that reflect the uncertainty in the diagnosis when uncertainty is high, for example, when the model consensus is close to 0.5, and make more confident decisions when it is close to 1. Also, clinicians can provide important pixels by conducting post hoc analyses such as Grad-CAM or Score-CAM [19,26].

### Limitations

This study acknowledges several limitations that may introduce bias into interpretations. Primarily, the ultrasonography datasets did not represent all ultrasonography devices worldwide. Given the variability in the device resolution, configuration, and manufacturer, classification performance cannot be universally applied. To mitigate this, we performed internal and external validations on various ultrasound devices and incorporated OOD data to achieve less biased and more widely applicable results. In addition, the implant images collected did not include all types of shells used worldwide; rather, we focused only on implants from 8 manufacturers licensed by the Ministry of Food and Drug Safety of the Republic of Korea. As a result, a multicenter study spanning multiple nations and including images of common implants in each region would allow for more generalized interpretations of the results. Lastly, as this research is at the feasibility stage, no existing studies have classified breast implant shell types. Consequently, it is challenging to compare other state-of-the-art methodologies. This limits the ability to assess the objectivity of this study’s findings or identify the best practice for classifying the shell types. However, as this is the first investigation into the classification of breast implant shell types, it can serve as the foundation for future studies in this area.

### Conclusion

The feasibility study presented demonstrates the potential of deep learning to accurately classify breast implant shell types from ultrasound images, addressing the current lack of standardized methods. Our findings underscore the importance of differentiating implant texture types, particularly for assessing the risk of BIA-ALCL. In addition, the adaptability of the deep learning model to account for imaging device variations and navigate prediction uncertainties opens promising avenues for robust, AI-driven clinical decision support in evaluating and managing breast implants.

## Appendix

Multimedia Appendix 1 Demographic characteristics of 1043 patients.

Multimedia Appendix 2 Model performance in the Canon dataset (D1) using 5-fold stratified cross-validation.

Multimedia Appendix 3 Example images of breast implants of the textured, smooth, and microtextured types.

## Abbreviations

AI: artificial intelligence
AUC: area under the curve
AUROC: area under the receiver operating characteristic curve
BIA-ALCL: breast implant–associated anaplastic large cell lymphoma
CNN: convolution neural network
DICOM: Digital Imaging and Communications in Medicine
FDA: Food and Drug Administration
Grad-CAM: Gradient-weighted Class Activation Mapping
MRI: magnetic resonance imaging
OOD: out-of-distribution
PACS: Picture Archiving and Communication System
PR: precision-recall
PRAUC: area under the precision-recall curve
ROC: receiver operating characteristic
XAI: explainable artificial intelligence
