# Supplementary material for: A Deep Learning Model to Predict Breast Implant Texture Types Using Ultrasonography Images: Feasibility Development Study
Source: JMIR Form Res. 2024 Nov 5;8:e58776. doi: 10.2196/58776 (PMC11576615; doi:10.2196/58776)
Supplement: Multimedia Appendix 1 [file formative_v8i1e58776_app1.docx]

# Multimedia Appendix 1

Table S1. Demographic characteristics of 1043 patients.

| Variables | Values (mean±SD) |
| --- | --- |
| Age (years old) | 37.9±10.0 |
| Sex (male-to-female ratio) | 0:1043 |
| Height (cm) | 162.4±5.1 |
| Weight (kg) | 52.0±6.4 |
| BMI (kg/m^2^) | 19.7±2.2 |

Table S2. Characteristics of surgery and breast implant of 1043 patients (2068 breast implants).

| **Variables** | **Values (%)** |
| --- | --- |
| **Surgery** | |
| Bilateral  Unilateral | 1,050 (99.1)  18 (0.9) |
| **Purpose of surgery** | |
| Aesthetic  Reconstructive | 2,035 (98.4)  33 (1.6) |
| **Chamber** | |
| Single  Dual | 2,068 (100)  0 |
| **Fill material** | |
| Silicone  Saline | 2,004 (96.9)  64 (3.1) |
| **Shell type** |  |
| Texture  Smooth | 403 (19.5)  1,665 (80.5) |
| **Manufacturer** | |
| HansBiomed Co., Ltd., Seoul, Korea    Groupe Sebbin SAS, Boissy-l’Aillerie, France    Mentor Worldwide LLC, Santa Barbara, CA, USA    Establishment Labs Holdings Inc., Alajuela, Costa Rica    Allergan plc, Dublin, Ireland    GC Aesthetics PLC, Apt Cedex, France    Polytech Health & Aesthetics, Dieburg, Germany    Silimed Inc., Rio de Janeiro, Brazil    Other | 594(28.7)  513(24.8)  257(12.4)  222(10.7)  215 (10.4)  85 (4.1)  56 (2.7)  22 (1.2)  104 (5.0) |
| **Surgery** | |
| Primary    Secondary  Rupture negative  Rupture present | 1,513 (73.2)    555 (26.8)  439 (21.2)  116 (5.6) |
| **Cause of Reoperation (n=265 case)** | **Values (%)** |
| Rupture  Baker III, IV capsular contracture  Dissatisfaction with shape  Fear of BIA-ALCL | 116 (43.8)  69 (26.0)  55 (20.8)  25 (9.4) |
