# Supplementary material for: A Deep Learning Model to Predict Breast Implant Texture Types Using Ultrasonography Images: Feasibility Development Study
Source: JMIR Form Res. 2024 Nov 5;8:e58776. doi: 10.2196/58776 (PMC11576615; doi:10.2196/58776)

# Multimedia Appendix 2

From stratified 5-fold cross-validation, our model showed an average AUROC of 0.98 and PRAUC of 0.88 in the Canon dataset captured with the Canon ultrasonography device (D1).

Figure S1. Model performance in the Canon dataset (D1) using 5-fold stratified cross-validation.


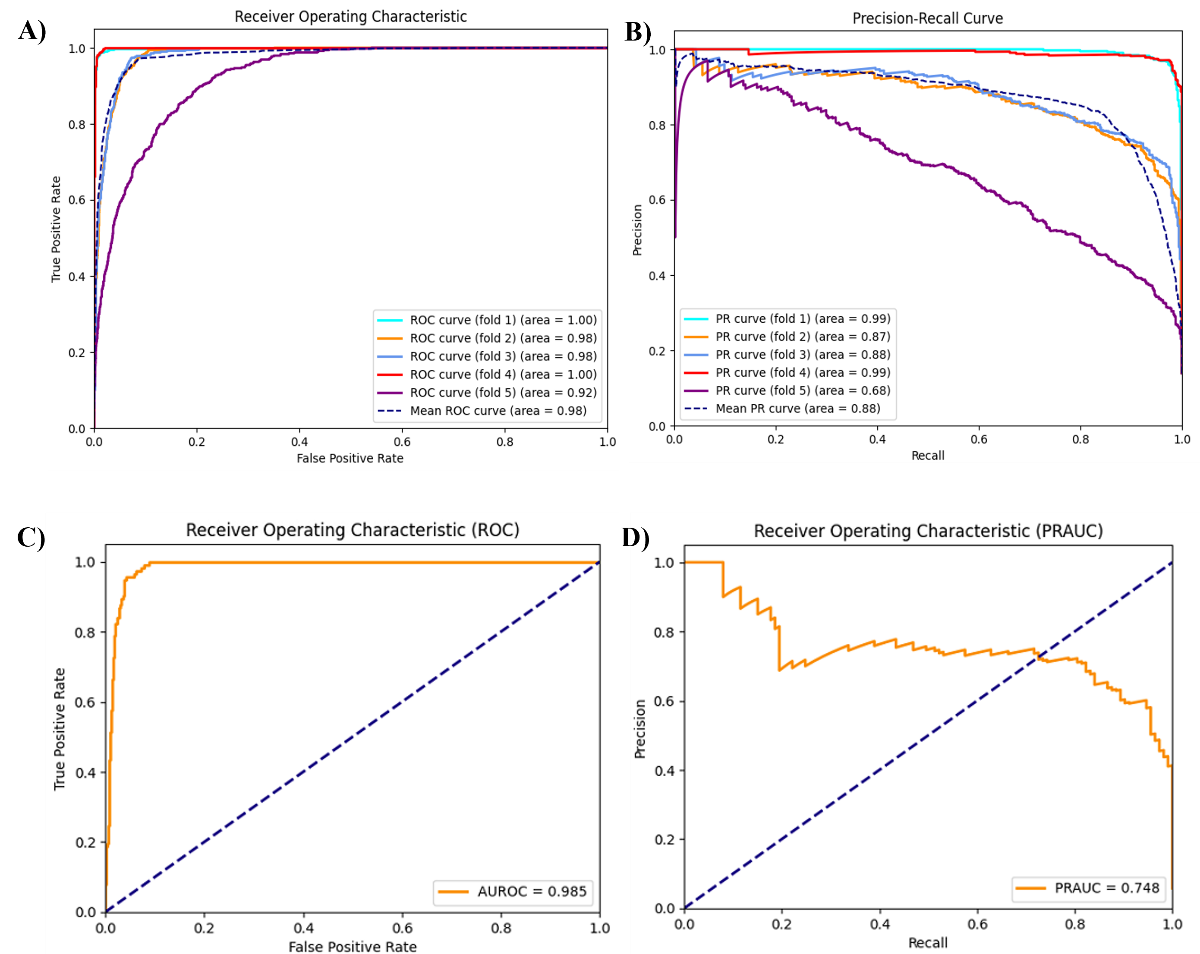

Supplement: Multimedia Appendix 2 [file formative_v8i1e58776_app2.docx]
