# Supplementary material for: A Deep Learning Model to Predict Breast Implant Texture Types Using Ultrasonography Images: Feasibility Development Study
Source: JMIR Form Res. 2024 Nov 5;8:e58776. doi: 10.2196/58776 (PMC11576615; doi:10.2196/58776)
Supplement: Multimedia Appendix 3 [file formative_v8i1e58776_app3.docx]

# Multimedia Appendix 3


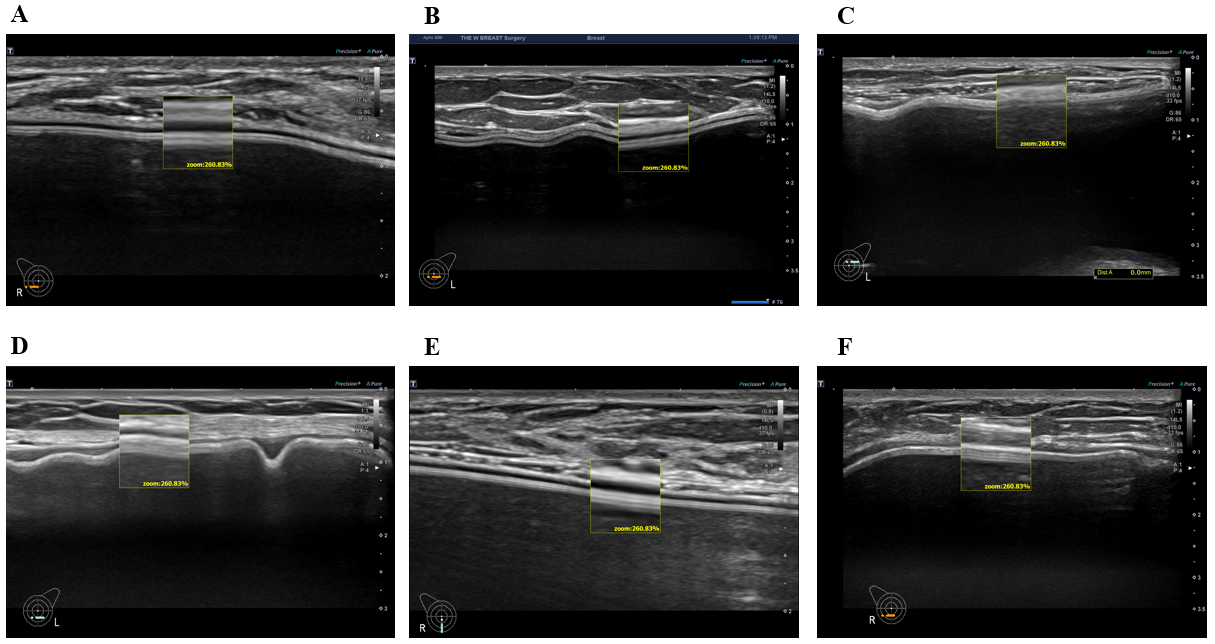


Figure S2. Example images of smooth type breast implants. A) Allergan smooth 3; B) Eurosilicone smooth; C) Mentor smooth 3; D) Sebbin smooth 2

Figure S1. Example images of texture type breast implants. A) Allergan Texture 5

B) Bellagle macro Texture 1; C) Eurosilicone Texture 1; D) Polytech Texture2; E) Sebbin Texture 1; F) Silimed texture 3


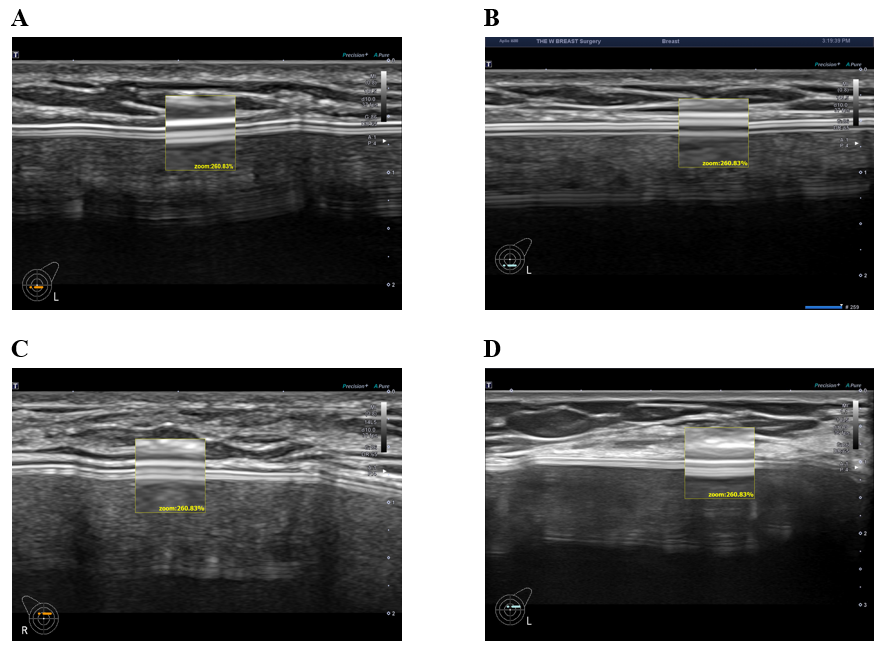


Figure S3. Example images of micro-texture type breast implants. A) Eurosilicone (GC Aesthetic); B) Hansbiomed Bellagel microtexture 3; C) Motiva microtexture; D) Sebbin microtexture

**
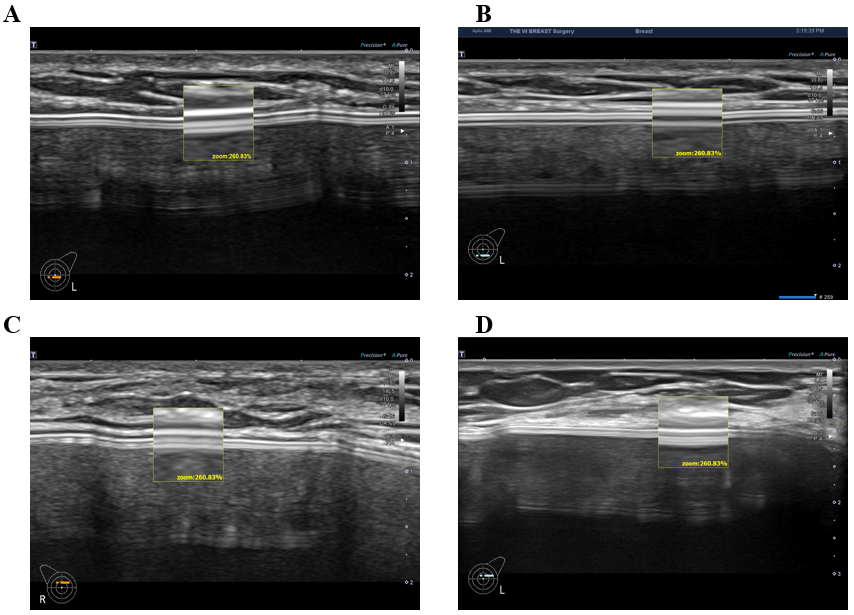
**
